# A large sustained endemic outbreak of multiresistant *Pseudomonas aeruginosa*: a new epidemiological scenario for nosocomial acquisition

**DOI:** 10.1186/1471-2334-11-272

**Published:** 2011-10-13

**Authors:** Cristina Suarez, Carmen Peña, Olga Arch, M Angeles Dominguez, Fe Tubau, Carlos Juan, Laura Gavaldá, Mercedes Sora, Antonio Oliver, Miquel Pujol, Javier Ariza

**Affiliations:** 1 IDIBELL, Infectious Diseases Service, Hospital Universitari de Bellvitge, University of Barcelona, Barcelona, Spain; 2 IDIBELL, Microbiology Service, Hospital Universitari de Bellvitge, University of Barcelona, Barcelona, Spain; 3 Microbiology Service, Hospital Son Espases, Palma de Mallorca, Spain; 4 IDIBELL, Preventive Medicine Service, Hospital Universitari de Bellvitge, University of Barcelona, Barcelona, Spain; 5 IDIBELL, Pharmacy Service, Hospital Universitari de Bellvitge, University of Barcelona, Barcelona, Spain; 6 Infectious Diseases Service, Hospital Universitari de Bellvitge, L'Hospitalet de Llobregat, Barcelona, Spain

## Abstract

**Background:**

Studies of recent hospital outbreaks caused by multiresistant *P.aeruginosa *(MRPA) have often failed to identify a specific environmental reservoir. We describe an outbreak due to a single clone of multiresistant (MR) *Pseudomonas aeruginosa *(PA) and evaluate the effectiveness of the surveillance procedures and control measures applied.

**Methods:**

Patients with MRPA isolates were prospectively identified (January 2006-May 2008). A combined surveillance procedure (environmental survey, and active surveillance program in intensive care units [ICUs]) and an infection control strategy (closure of ICU and urology wards for decontamination, strict compliance with cross-transmission prevention protocols, and a program restricting the use of carbapenems in the ICUs) was designed and implemented.

**Results:**

Three hundred and ninety patients were identified. ICU patients were the most numerous group (22%) followed by urology patients (18%). Environmental surveillance found that 3/19 (16%) non-ICU environmental samples and 4/63 (6%) ICU samples were positive for the MRPA clonal strain. In addition, active surveillance found that 19% of patients were fecal carriers of MRPA. Significant changes in the trends of incidence rates were noted after intervention 1 (reinforcement of cleaning procedures): -1.16 cases/1,000 patient-days (95%CI -1.86 to -0.46; p = 0.003) and intervention 2 (extensive decontamination): -1.36 cases/1,000 patient-days (95%CI -1.88 to -0.84; p < 0.001) in urology wards. In addition, restricted use of carbapenems was initiated in ICUs (January 2007), and their administration decreased from 190-170 DDD/1,000 patient-days (October-December 2006) to 40-60 DDD/1,000 patient-days (January-April 2007), with a reduction from 3.1 cases/1,000 patient-days in December 2006 to 2.0 cases/1,000 patient-days in May 2007. The level of initial carbapenem use rose again during 2008, and the incidence of MRPA increased progressively once more.

**Conclusions:**

In the setting of sustained MRPA outbreaks, epidemiological findings suggest that patients may be a reservoir for further environmental contamination and cross-transmission. Although our control program was not successful in ending the outbreak, we think that our experience provides useful guidance for future approaches to this problem.

## Background

Classically, *P.aeruginosa *nosocomial infections have been considered to be polyclonal endemic infections that follow secondary endogenous intestinal and primary respiratory tract colonization in patients admitted to ICUs or other hospital wards who have previously received antibiotic therapy [1-3]. However, in this setting, outbreaks caused by some particularly transmissible strains which often show multiresistance to antibiotics have also been reported. The capacity to control these outbreaks varies greatly and dependes on the reservoir. Outbreaks are usually circumscribed in time and attributable to a point source of infection which can be identified in the environment [4-8].

Over the past decade, an increase in hospital outbreaks caused by multiresistant *P.aeruginosa *(MRPA) has been reported in a new epidemiological setting. Studies have often failed to identify a specific environmental reservoir [9-15], and point toward patients as an additional potential reservoir, as in the case of a large sustained epidemic/endemic caused by multiresistant *Acinetobacter baumannii *[16]. While several studies of the risk factors and clinical features of patients colonized or infected with multiresistant *P.aeruginosa *have been conducted [17,18], the management strategies for these prolonged MRPA outbreaks and the contribution of antibiotic restriction [9] to their control have not been analyzed in depth.

Since 2003 there has been an increase in the number of carbapenem-resistant *P.aeruginosa *isolates in our hospital. A retrospective microbiological revision in 2005 showed a progressive increase in *P.aeruginosa *strains exhibiting the same multiresistant antibiotype, and molecular typing of selected strains demonstrated the presence of an outbreak in several hospital wards. Despite strict barrier precautions for controlling infection, the incidence of MRPA continued to rise throughout the hospital.

Here, we describe a large-scale sustained outbreak of a multiresistant strain of *P.aeruginosa *in our hospital and evaluate the effectiveness of the control measures applied.

## Methods

### Setting and Definitions

The study was performed at the Hospital Universitari de Bellvitge, a 900-bed public tertiary-care institution for adult patients. The MRPA surveillance programme was initiated in January 2006 and all patients colonized or infected with MRPA were prospectively identified.

MRPA was defined as strains resistant to ≥ 3 of the following classes of antibiotics: antipseudomonal penicillins, antipseudomonal oxyimino-β-lactams, fluoroquinolones, aminoglycosides, and carbapenems [19]. A clinical sample positive for MRPA was considered to have been acquired in a specific ward if it appeared either during a stay in this ward or within a week of discharge from it. The remaining patients with clinical samples positive for *P.aeruginosa *and without the MRPA phenotype isolate from the time of admission to the time of discharge from hospital were considered non-MRPA.

Clinical assessment was determined in accordance with the definitions of the Centers for Disease Control and Prevention for nosocomial infections [20]. Patients with positive clinical samples for MRPA but without related signs or symptoms of infection were considered to be colonized.

### Microbiological studies

*P. aeruginosa *strains were identified and tested for antimicrobial susceptibility by a MicroScan automated microdilution system using CN1S and CO1S panels (Dade International, West Sacramento, CA, USA). CLSI (Clinical and Laboratory Standards Institute) criteria [21] were used to define susceptibility or resistance to these antimicrobial agents.

We selected 284 MRPA phenotype strains isolated throughout the whole period (2006 [132], 2007 [101], 2008 [51]) for pulsed-field gel electrophoresis (PFGE) analysis using a method described previously [13]. DNA was digested with *Spe*I and DNA fragments were separated with a CHEF DR III apparatus (Bio-Rad Laboratories, Hercules, CA, USA). Electrophoresis was run at 6 v/cm, 14°C for 20 hours with pulses ranging from 0,5 to 25 seconds. DNA restriction patterns generated by PFGE were interpreted according to the guidelines [22]. Isolates with PFGE patterns differing in more than four fragments were ascribed to distinct genotypes; differences in four restriction fragments or fewer were considered to be subtypes of a single genotype [23].

The mechanisms responsible for the multidrug-resistant phenotype detected were studied in three representative isolates from the epidemic clone. The potential presence of horizontally acquired β-lactamases was explored by phenotypic tests, which included Etest MBL strips (AB Biodisk, Solna, Sweden) for the detection of class B carbapenemases and double disk synergy tests (DDST) for the detection of ESBLs, using amoxicillin-clavulanate and ceftazidime, cefepime, aztreonam, imipenem, or meropenem disks (distance from 5 to 30 mm). To examine the involvement of mutation-driven resistance mechanisms in the multidrug-resistant phenotype, the relative mRNA expression of the genes encoding the major *P. aeruginosa *efflux pumps (*mexB*, *mexD, mexF *and *mexY*) and the chromosomal cephalosporinase (*ampC*) was determined by real-time PCR as described elsewhere [24,25]. Isolates were considered to be hyperproducers if the relative expression of the corresponding gene was at least three times (*mexB*) or ten times (*mexD*, *mexF mexY*, and *ampC*) greater than the figure documented for the reference PAO1 strain. In addition, in order to detect inactivating mutations causing carbapenem resistance, *oprD *genes from these isolates were amplified by PCR and fully sequenced, using previously described primers and conditions [26]. The sequences obtained were compared with those available in the GenBank.

### Environmental survey

To determine the possible route of transmission or reservoir in the hospital's physical environment, a total of 78 samples, 30 from room equipment (bedside tables, bed rails, phones, plug holes, tap handles, door handles, call buttons), 36 from sink surfaces, and 12 from water samples were taken from the urology and ICU wards, the wards which initially had the highest incidence. Twenty samples, (13 from urology wards and seven from the ICU), were obtained in February 2006 using moistened swabs, another 16 samples were obtained in September 2006 from the urology wards, and the remaining 42 samples were collected from the ICU during 2007. In addition, to improve our capacity to detect contamination, in 2008 we modified the swab technique by introducing the use of moistened sterile gauze pads [27]. Another 82 samples were collected (63 from ten ICU rooms and 19 from four non-ICU rooms). All these samples were taken from room equipment. During the surveillance procedure, all the non-ICU rooms had admitted patients with known MRPA colonization, compared with only three ICU rooms. The genetic similarity of MRPA strains isolated in the environmental survey was investigated by PFGE.

### Active surveillance program

To determine the strength of the human reservoir, we carried out longitudinal active surveillance of rectal swabs in an ICU unit over a one-month period (March 2006). Weekly rectal swab samples were obtained on admission in order to identify digestive tract carriage of MRPA between ICU admission and discharge or the time to MRPA clinical sample detection. Patients admitted to the unit for less than 48 hours and those with MRPA digestive tract colonization at ICU admission were excluded.

### Infection control interventions

Infection control measures: Intervention 1: Disposable aprons and gloves were used while caring for MRPA colonized/infected patients, and cleaning procedures were strictly supervised by infection control nurses (rooms were routinely cleaned once a day using water and liquid soap with 500 parts per million [ppm] hypochlorite, and rooms with colonized patients were cleaned twice a day in the same way but with a hypochlorite concentration of 1,000 ppm, and using a specific checklist for high-touch surfaces). ICU patients were in single rooms and patients in non-ICU wards were spatially segregated inside a single room. These infection control measures were introduced in February 2006. Intervention 2: Environmental cleanliness was reinforced, particularly in the ICU (which was closed for extensive decontamination, consisting in rigorous cleaning of the medical equipment and painting of the wards, in July-August 2006 and July-August 2007) and the urology ward (which was decontaminated in August 2007).

Restriction of carbapenem use in ICUs: In January 2007 the use of carbapenem for ICU patients was restricted. Compliance with this policy was monitored. Consumption was expressed as defined daily doses (DDD) [28].

### Statistical analysis

An interrupted time series analysis was performed using the segmented linear regression procedure [29], evaluating the impact of the intervention on the level (abrupt change immediately following the intervention) and the secular trend of the series (change in the slope of the incidence rate following the intervention). The level and trend of the pre-intervention segments served as the control for the post-intervention segment.

MRPA patients included those who presented at least one MRPA specimen obtained during admission; previously known cases who tested positive on readmission were excluded, and newly identified cases attending the outpatient clinic were included from the date of prior admission. Colonization pressure was calculated monthly in the ICUs and the urology ward as follows: number of MRPA patient-days × 100/total number of patient-days [30].

**Figure 1:**
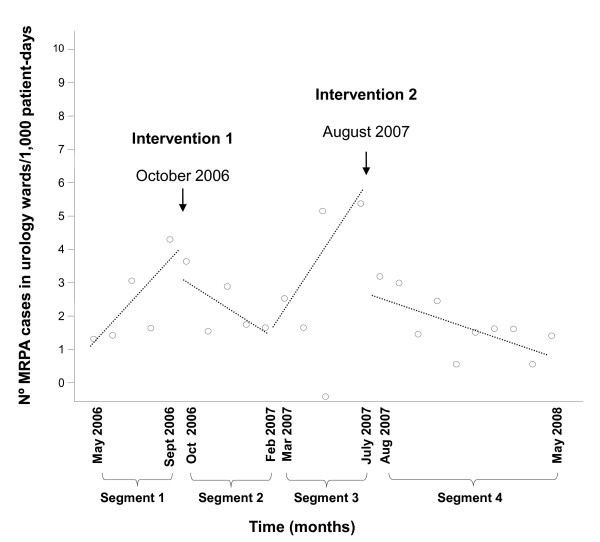
**Reduction in the rate of MRPA cases in urology ward: Intervention 1: reinforcement cleaning procedures; Intervention 2: extensive decontamination**.

To evaluate the effect of the interventions performed in the urology ward, four time segments were defined (Figure 1). Segments 1 and 3 served as the pre-intervention (baseline) periods. Time points before May 2006 were excluded from the analysis, since there were fewer than three observations before and after an intervention performed in February 2006 [31]. The interventions studied were: Intervention 1 "reinforcement of control measures and cleaning procedures" (October 2006) and Intervention 2 "extensive decontamination" (August 2007). The analysis was adjusted for the colonization pressure of the immediately preceding month and for the level and trend of the pre-intervention periods. Statistical analysis was performed with SPSS software, version 14.0.

### Ethical considerations

The study formed part of the medical care provided in the setting of the surveillance and control of nosocomial outbreaks, and the physician had good reason to believe that participation in the research study would not adversely affect the health of the patients. All the samples and exams performed on patients were part of standard care procedures for epidemiological control. The study was approved by the local ethic committee of the hospital Universitari de Bellvitge and no informed consent was requested.

## Results

### Description of the outbreak

From January 2006 to May 2008, 390 consecutive patients were colonized or infected by a main phenotype of MRPA; 288 (74%) patients were males with a mean age of 62.4 (SD ± 19.7) years; 85 (22%) were ICU patients and 305 patients acquired the MRPA strain in a non-ICU setting. The urology service [54 patients/305 (18%)] was the non-ICU ward with the highest rates of MRPA acquisition; the remaining patients were recorded in 30 other wards.

Among the 390 patients with clinical samples for MRPA, 165 (42%) patients were infected, with urinary tract infection [45 episodes (28%)] being the most frequent MRPA infection. Forty-six episodes (28%) were intra-abdominal infections: 36 (22%) surgical site infections, and 10 (6%) biliary infections. All 37 (23%) episodes of lower respiratory tract infection were observed in ICU patients. The remaining infections were: eleven (7%) primary bloodstream infections, 12 (7%) bone and joint infections, nine (6%) soft-tissue infections and five miscellaneous infections. Seventy-two (44%) out of 165 infected patients died. Of these deaths, 14 (19%) were attributed to MRPA infection.

### Microbiological and genotypic analysis

The antibiotic susceptibility patterns of MRPA strains isolated during the study period are summarized in Table 1. Genotypic analysis showed that the selected MRPA strains belonged to a single clone responsible for a large sustained outbreak and no relationship was found between this majority clone and the MRPA strains detected in our hospital in an earlier MRPA outbreak [5].

**Table 1:** Antibiotic susceptibility of multiresistant *P.aeruginosa *clone strains

	Range	MIC_50_	MIC_90_	Breakpoint		%I^a^	%R^b^	Breakpoint		%I^a ^	R^b^
				CLSI 2009				EUCAST2009			
				S	R			S	R		
PIP	≤ 16- > 64	64	> 64	≤ 64	≥128	-	39	≤ 16	> 16	-	97
TIC	≤ 16- > 64	64	> 64	≤ 64	≥128	-	42	≤ 16	> 16	-	98
TZP	≤ 16/4- > 64/4	64/4	> 64/4	≤ 64/4	≥128/4	-	35	≤ 16/4	> 16/4	-	93
ATM	2- > 16	16	> 16	≤ 8	≥32	85	14	≤ 1	> 16	85	14
CAZ	2- > 16	16	> 16	≤ 8	≥32	75	22	≤ 8	> 8	-	97
FEP	2- > 16	16	> 16	≤ 8	≥32	82	17	≤ 8	> 8	-	99
GEN	≤ 4- > 8	> 8	> 8	≤ 4	≥16	0	99	≤ 4	> 4	-	99
TOB	≤ 4- > 8	> 8	> 8	≤ 4	≥16	1	95	≤ 4	> 4	-	96
AMK	≤ 8- > 16	≤ 8	> 16	≤ 16	≥64	0	21	≤ 8	> 16	29	21
IPM	8- > 8	> 8	> 8	≤ 4	≥16	10	90	≤ 4	> 8	10	90
MEM	8- > 8	> 8	> 8	≤ 4	≥16	33	67	≤ 2	> 8	33	67
CIP	2- > 2	> 2	> 2	≤ 1	≥4	2	98	≤ 0.5	> 1		100
Colistin	≤ 2	≤ 2	≤ 2	≤ 2	≥4	0	0	≤ 2	> 2	0	0

^a ^Intermediate.

^b ^Resistant.

PIP = Piperacillin; TIC = Ticarcillin; TZP = Piperacillin-tazobactam; ATM = Aztreonam; CAZ = Ceftazidime; FEP = Cefepime; GEN = Gentamicin; TOB = Tobramycin; AMK = Amikacin; IPM = Imipenem; MEM = Meropenem; CIP = Ciprofloxacin.

All phenotypic tests for the detection of acquired β-lactamases yielded negative results. In contrast, the pan-β-lactam resistance phenotype was found to be driven by the interplay of the hyperproduction of the chromosomal cephalosporinase AmpC (238 ± 34-fold increased expression of *ampC *respect to wild-type reference strain PAO1) and the inactivation of OprD porin, caused by a C to T mutation in nucleotide 424 of *oprD*, which leads to a premature stop codon at amino acid 142. Furthermore, no significant modification of the expression of the efflux pump encoding genes *mexB, mexD, mexF, or mexY *was detected.

### Environmental survey results

Three of 36 (8%) initial samples were positive for clonal MRPA strain. All three strains were isolated in wet areas in the urology ward. None of the remaining 42 samples (moistened surfaces, room equipment and water samples) from the ICU wards showed MRPA growth. Thirteen samples, five of them from water, showed growth for carbapenem-susceptible *P.aeruginosa*.

In addition, with the modified gauze technique [27], three of 19 (16%) samples from non-ICU rooms were positive for the MRPA clonal strain, while only four of 63 (6%) ICU samples were positive.

### Active surveillance program

During the study period, 42 patients were admitted to the ICU; of these, six patients with an ICU stay of less than 48 hours and five carriers of digestive tract MRPA at ICU admission were excluded. Thirty-one patients were included, and six digestive tract carriers were colonized by MRPA (19%). The mean time to MRPA acquisition between ICU admission and digestive tract carriage was 22 days. The mean time between carriage of MRPA in the digestive tract and the detection of positive clinical samples was 11 days (range 1 to 21 days).

### Response to infection control intervention programs

Before the interventions, there was a significant month to month increase in MRPA cases in the urology ward: segment 1: increase of 0.67 cases/1,000 patient-days (95%CI 0.21 to 1.12; p = 0.006) and segment 3: increase of 1.53 cases/1,000 patient-days (95%CI 0.84 to 2.23; p < 0.001). However, significant changes in the trends of incidence rates were noted after both interventions: intervention 1: -1.16 cases/1,000 patient-days (95%CI -1.86 to -0.46; p = 0.003) and intervention 2: -1.36 cases/1,000 patient-days (95%CI -1.88 to -0.84; p < 0.001). This represents a mean decrease of 5.4 MRPA cases/1,000 patient-days after intervention 1, and an even more marked reduction, 8.4 cases/1,000 patient-days, after intervention 2. Concomitantly, after intervention 2, an immediate effect was found in the first month: -1.58 cases/1,000 patient-days (95%CI -3.50 to 0.33; p = 0.09) (Figure 1).

**Figure 2:**
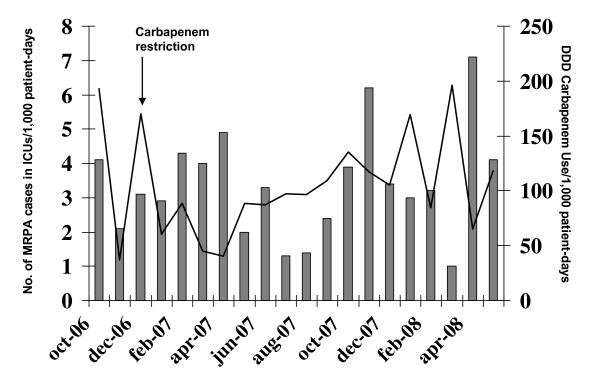
**MRPA incidence rates in ICUs during and after the implementation of the restricted use of carbapenems**. Bars: incidence rates (n° MRPA cases/1,000 patient-days). Line: DDD carbapenem use/1,000 patient-days.

In addition, restricted use of carbapenems was initiated in January 2007 (Figure 2). Carbapenem use decreased from 190-170 DDD/1,000 patient-days in the period immediately before the implementation of this restriction (October-December 2006) to 40-60 DDD/1,000 patient-days after its initiation (January-April 2007), with a reduction from 3.1 cases/1,000 patient-days in December 2006 to 2.0 cases/1,000 patient-days in May 2007. However, after this strong initial decrease, we observed a moderate increase in carbapenem consumption, which rose to around 100 DDD/1,000 patient-days from May to December 2007 and a progressive increase in incidence. Segmented linear regression analysis could not easily be applied in ICU intervention (carbapenem use restriction) since no evident linear patterns were found following the intervention.

## Discussion

We provide epidemiological information on a large-scale sustained MRPA outbreak occurring at our hospital over a period of more than three years. An epidemic clone prevailing in the urology and ICU wards spread rapidly throughout many other wards despite an epidemiological surveillance program; no specific source of outbreak could be identified and the clone became endemic. This is a new epidemiological scenario with serious consequences.

Our observations contrast strongly with the classical view of the epidemiology of nosocomial *P.aeruginosa *infections. These nosocomial *P.aeruginosa *infections were considered to be polyclonal, endemic and opportunistic, affecting patients with underlying diseases undergoing multiple manipulations and receiving broad spectrum antibiotic therapy [3].

Several hospital outbreaks caused by multiresistant *P.aeruginosa *have been reported [9-15]. Although colonization by *P.aeruginosa *frequently precedes overt infection, the original source of the organism and the precise mode of transmission are often unclear.

In our large clonal endemic setting, we were unable to identify a particular reservoir responsible for the outbreak. We investigated all the wet areas that were likely to be sources of contamination. Environmental surveillance was performed in the urology and ICU wards, the wards with the highest incidence, but in neither case did the study provide a great deal of information. The level of detection improved when a moistened gauze was used (0% in ICU and 8% in non-ICU using swabs versus 6% in ICU and 18% in non-ICU with gauze), although no parallel analysis of the two techniques was performed. In addition, the sites that tested positive for the epidemic clone were near colonized patients, and the presence of this clone in the environment may have been a simple consequence of the outbreak rather than a source for transmission of the strain [32]. Nevertheless, two specific interventions involving the reinforcement of cleaning procedures for extensive decontamination were applied in the urology wards and MRPA rates decreased significantly during the months following the interventions, a finding that supports the transitory efficacy of these epidemiological control measures

The results of our active surveillance program and environmental cultures in the ICU setting suggest that patients may indeed be a reservoir in the maintenance of the monoclonal MRPA outbreak. Our capacity of detection of MRPA intestinal carriers in ICUs was lower than the rates found in the screening programs of two other outbreaks at our hospital [33,34]; however, in those two settings the capacity of the outbreak to spread was higher. These findings are borne out by the longer period of time recorded between ICU admission and digestive colonization: 22 days in the present study versus < 7 days in those outbreaks.

No doubt, cross-transmission plays a relevant epidemiological role in our MRPA ICU patients [35], but as no hand-print cultures of health care workers were performed in our study, we cannot provide direct evidence that strains were transmitted patient to patient by the health care workers.

Moreover, our data suggest that antibiotic pressure plays a decisive role, by altering the ecological niche in these patients and providing a selective growth advantage for MRPA organisms. Thus, given that the carbapenem consumption in our ICUs in 2006 was high, we assumed that carbapenem restriction might make a significant contribution to controlling the outbreak. The carbapenem restriction program carried out during 2007 was initially followed by a marked reduction; however, a moderate increase was recorded in the second semester and the levels rose again during 2008. Concomitantly, an increase in the level of piperacillin-tazobactam use was observed and a new cluster, particularly in the ICU, was found [36]; the dominant use of this antibiotic could have favored the emergence and spread of this different clone, and as a result, the carbapenem restriction was relaxed. Carbapenem restriction did not significantly reduce the number of MRPA cases, although this antibiotic program may not have been sufficiently rigorous or prolonged. Furthermore, we cannot rule the possibility that the use of fluoroquinolones may also have contributed to promoting these MRPA strains [17]. Although fluoroquinolone consumption did not increase during the period analyzed (data not shown), overall fluoroquinolone use was considerably higher than that of other antipseudomonal antibiotics. Thus, the limitation of fluoroquinolone use should have been included in our antibiotic restriction program.

Finally, our epidemiological control program had several limitations. First, we did not perform decontamination in other hospital wards; second, it is possible that the evaluation of MRPA digestive tract carriers in certain non-ICU wards would have allowed earlier spatial segregation and would thus have prevented a substantial number of possible cross-transmissions; and third, an extensive antibiotic program restricted to non-ICU wards might have contributed to lower incidence rates.

## Conclusion

In conclusion, we faced a large and sustained MRPA outbreak that became endemic in our hospital after three years. In the absence of a recognized source of contamination, the reasons for the wide dissemination of the clone remain unclear. However, considering the general epidemiology of nosocomial *P.aeruginosa *infections, we speculate that more efficient nosocomial clones are more likely to acquire resistance determinants and that antibiotic pressure in the hospital environment favors their further spread [37]. Epidemiological findings suggest that patients may be a reservoir for further environmental contamination and cross-transmission. However, although unlikely, we cannot completely rule out the possibility of an environmental reservoir. Further studies should try to determine the extent to which realistic and prolonged restriction antibiotic programs can contribute to fighting this epidemiological challenge.

## Competing interests

The authors declare that they have no competing interests.

## Authors' contributions

CS participated in recruitment of subjects, performed the statistical analysis and drafted the first version of the manuscript. CP was the study's principal investigator, and participated in its design and coordination, supervision of analyses, and drafting. OA participated in the collection of epidemiologic data. MAD performed the PFGE analysis. FT performed the phenotype analysis. CJ studied the mechanisms responsible for the multidrug-resistant phenotype. LG participated in the design and statistical analysis. MS calculated antimicrobial consumption. AO helped to interpret the results of mechanisms of resistance. MP participated in the design and in the analysis of results. JA participated in design and provided expert oversight. All authors read and approved the final manuscript.

## Pre-publication history

The pre-publication history for this paper can be accessed here:

http://www.biomedcentral.com/1471-2334/11/272/prepub
